# Terahertz-frequency plasmonic-crystal instability in field-effect transistors with asymmetric gate arrays

**DOI:** 10.1038/s41598-024-62492-3

**Published:** 2024-05-24

**Authors:** G. R. Aizin, S. Mundaganur, A. Mundaganur, J. P. Bird

**Affiliations:** 1 Kingsborough College & the Graduate Center of the City University of New York, Brooklyn, NY 11235 USA; 2 Department of Electrical Engineering, University at Buffalo, the State University of New York, Buffalo, NY 14260-1900 USA

**Keywords:** Electrical and electronic engineering, Other photonics

## Abstract

We present a theory for plasmonic crystal instability in a semiconductor field-effect transistor with a dual grating gate array, designed with strong asymmetry in the elementary cell of this “crystal”. We demonstrate that, under the action of a dc current bias, the Bloch plasma waves in the plasmonic crystal formed in this transistor develop the Dyakonov–Shur instability. By calculating the energy spectrum and instability increments/decrements—which govern the growth/decay of excitations within the plasmonic crystal—we analyze the dependence of the latter on the electron drift velocity and the extent of the structural asymmetry. In contrast with the corresponding problem for gate arrays with symmetric unit cells, the presence of finite plasma instability increments across the entire Brillouin zone is established. This important difference points to the possibility of exciting sustained, radiating, non-linear electron plasma oscillations in the instability endpoint of the asymmetric array. These structures should be readily implementable in common semiconductor heterostructures, using standard nanofabrication techniques, enabling operation at room temperature. Long-range coherence of the unstable plasma oscillations, generated in the elementary cells of the crystal, should dramatically increase the radiated THz electromagnetic power, making this approach a promising pathway to the generation of THz signals.

## Introduction

The design of efficient tunable on-chip sources of terahertz (THz) electromagnetic (EM) radiation remains a challenging technological problem. Such sources are one of the key required elements of nanoscale, beyond-6G, wireless communications systems, with potential applications ranging from wireless networks-on-chip^1^ to biomedical systems^2^. All-electronic on-chip THz sources include Schottky diode frequency multipliers^3^, GaN IMPATT (impact ionization avalanche transit-time diode) diodes^4^, and resonant tunneling diodes^5^, all of which operate at the lower end of the THz spectrum (~ 0.1–1 THz)^6^, and plasmonic THz field-effect transistors (TeraFETs) with the capability to operate at frequencies up to 10 THz^7^.

Plasmonic TeraFETs use the instability of collective plasma oscillations in the two-dimensional (2D) electron channel of a transistor to generate EM radiation. The channel functions as a plasmonic cavity, reflecting plasma waves at its opposite ends. As first predicted by Dyakonov and Shur^8^, when the reflection conditions differ substantially, a constant (dc) drive current may induce plasma instability. Recently, it was also demonstrated numerically, for both compound semiconductor- and graphene-based systems, that the final stationary state developed at the instability endpoint represents a nonlinear anharmonic plasmonic oscillator, with a fundamental frequency that is close to that of the original unstable plasma mode^9–13^. Since these frequencies lie in the THz range and are easily tunable via the gate voltage of the transistor, plasmonic TeraFETs are potentially attractive for a variety of applications that may utilize the THz EM radiation generated in this process.

Previous experimental studies have demonstrated signatures of the Dyakonov–Shur (DS) instability in transistor-based plasmonic cavities and have also revealed the presence of THz EM radiation^14,15^. Recently, transistors with specially engineered structural asymmetry have been implemented and features characteristic of the DS instability were found in their transistor characteristics at temperatures as high as 350 K^16^. In spite of this, the radiated EM power measured in previous experiments^14,15^ has been too small (~ nW) to enable practical applications. To improve the emitted power of such radiation transistors can be combined into arrays^17^, but the lack of coherence among plasma oscillations, generated in the individual devices, impedes any significant power increase.

The most promising approach to increase the radiated power from plasmonic sources is to design transistors in which a suitable periodic modulation of the electron channel is introduced. This idea has long motivated the study of transistors with “grating-gate” geometries^18^ and has more recently been proposed in the context of a plasmonic transistor with periodically modulated channel width^19^. Periodic modulation of the 2D electron channel leads to the formation of a plasmonic crystal, with an energy spectrum that was first described theoretically in^20^ and confirmed experimentally in^21,22^. In such a system, plasma oscillations in the individual elementary cells are coupled to one another via EM interaction, and the coherence of these oscillations results in the formation of a Bloch plasma wave across the entire crystal. Under such conditions, once plasma waves become unstable in the individual elementary cells, a drastic enhancement of the radiated EM power should occur due to coherent addition of the EM wave amplitudes generated by the different cells^19,23,24^, leading to total radiated EM power proportional to *N*^2^ where *N* is the number of elementary cells.

In spite of the many studies that have addressed the issue of plasmon generation in transistors incorporating different gate-array geometries^18–27^, the need for practical schemes for on-chip THz-signal generation nonetheless remains. In this paper, we consider a high-electron-mobility transistor with an asymmetric grating-gate array that generates a periodically modulated (equilibrium) carrier density in the transistor channel. In contrast with previous studies^27^, we consider the case of strong modulation of the electron density, yielding well-separated frequencies for electron plasma oscillation in the gated and ungated sections of the transistor channel. This then allows us to focus on the situation in which plasma oscillations are excited in the individual elementary cells defined by the gates. These oscillations are coupled to form a plasmonic crystal, with the ungated sections providing coherent electromagnetic linkage between the gated plasmonic cavities. The asymmetric boundary conditions necessary for the plasma instability in individual cells are provided by placing a narrow metal finger near one edge of each gated cavity (dual grating gate) as explained in detail below. This array structure is designed to achieve strong asymmetry within the elementary cells of the resulting plasmonic crystal, together with the coherence of plasma oscillations in individual cells. We present a quantitative theoretical study of plasma-wave generation in the channel of such a transistor and demonstrate that a dc bias current may cause the plasma band modes of this periodic system to become unstable within the *entire* Brillouin zone. In this way, we identify the conditions under which, with dc current biasing, the plasma-wave instability increment can exceed the damping rate of the plasma waves due to (phonon and impurity) scattering. In these circumstances, any spontaneous charge inhomogeneity that develops in the channel will grow exponentially with time as plasma waves are excited. This process should eventually lead to sustained plasma oscillations once the initial instability reaches its endpoint, allowing the transistor to serve as a source of THz EM radiation^9–13^. (Note that our analysis involves the linear response of the system only. Full solution of the nonlinear hydrodynamic equations, together with the system of Maxwell’s equations, would be needed to determine the true instability endpoint that may result in the emission of THz electromagnetic radiation by the plasmons^11–13^. This problem is beyond the scope of this paper and will be considered in a separate study.)

## Results

### Theoretical formulation

**Figure 1:**
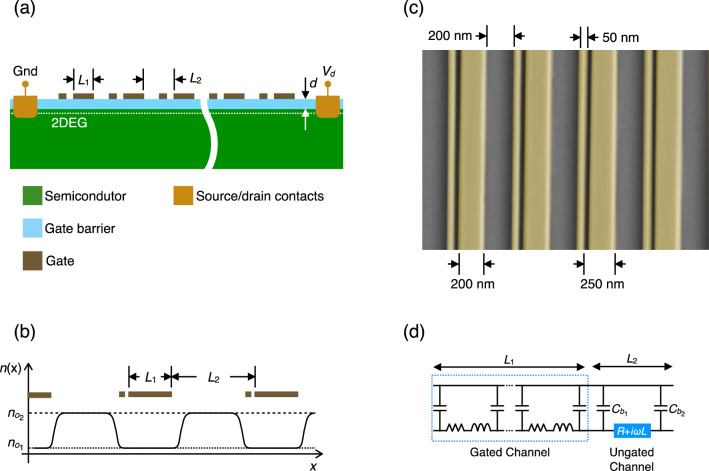
(**a**) Cross sectional schematic showing the basic structure of the suggested grating-gate device. (**b**) Density profile induced in the device by biasing the wide gates. (**c**) False-color electron micrograph showing a grating-gate fabricated on a GaAs substrate. Metal gates are Cr/Au (5–50 nm). (**d**) Equivalent electric circuit diagram of the device of panels (**a**–**c**).

The transistor design that we propose is shown schematically in Fig. 1a. The channel of this device consists of periodically spaced, gated and ungated, sections, of respective lengths $L_{1}$ and $L_{2}$. Each cell of the array comprises a pair of dissimilar gates, the wider of which is biased to generate the periodically modulated density profile shown in Fig. 1b. In the discussion that follows, we denote the equilibrium electron density in the gated and ungated sections as $n_{01}$ and $n_{02}$, respectively ($n_{01} \ll n_{02}$). The narrow gate within each cell is considered to be unbiased, with its purpose being to produce an additional capacitive link^27^ between the 2D electron channel and one of the edges of the wider gate (the left edge as shown in Fig. 1a). This narrow element is therefore the source of the asymmetry that is required to achieve plasma-wave instability in the plasmonic cavities formed under the wide gates. In Fig. 1c we demonstrate the feasibility of realizing the periodic gate geometry via nanolithography.

When electron–electron interactions are the dominant source of scattering within the system, plasmonic excitations in the gated sections of the 2D electron channel can be described in the hydrodynamic approximation. Neglecting the influence of random electron scattering (i.e., assuming that $\omega \tau \gg 1$ where $\omega$ is the plasma frequency and $\tau$ is momentum relaxation time), the relevant hydrodynamic equations for the local electron-fluid density ($n\left( {x,t} \right)$) and velocity ($v\left( {x,t} \right)$) are the continuity equation and the Euler equation:

1 $$\frac{\partial n}{{\partial t}} + \frac{{\partial \left( {nv} \right)}}{\partial x} = 0 , \frac{\partial v}{{\partial t}} + v\frac{\partial v}{{\partial x}} = \frac{e}{{m^{*} }}\frac{\partial \varphi }{{\partial x}}$$

Here, $\varphi \left( {x,t} \right)$ is the electric potential in the channel, and $- e$ and $m^{*}$ are the electron charge ($e$ = 1.602 × 10^−19^ C) and effective mass, respectively.

To solve (1), we linearize it assuming small fluctuations of the electron density ($n = n_{01} + \delta n$) and velocity ($v = v_{0} + \delta v$, where $v_{0}$ is the electron drift velocity in the plasmonic cavity with constant source-drain current). In the gated sections of the channel, fluctuations of the electron density and the electric potential ($\delta \varphi$) are connected via $- e\delta n = C\delta \varphi$, where $C = \varepsilon \varepsilon_{0} /d$ is the capacitance per unit area between the channel and gate, and *d* and $\varepsilon$ are the thickness and dielectric constant, respectively, of the gate barrier (see Fig. 1a). In this quasistatic limit, solution of the linearized form of Eq. (1), for small fluctuations of $\delta n_{\omega }$ and $\delta v_{\omega }$, can be written as^28^:

$$I_{\omega } \left( {x,t} \right) = \left( {I_{1} e^{{ - iq_{1} x}} + I_{2} e^{{ - iq_{2} x}} } \right)e^{i\omega t} ,$$

2 $$V_{\omega } \left( {x,t} \right) = \frac{1}{CW}\left( {\frac{{I_{1} }}{{v_{0} + v_{p} }}e^{{ - iq_{1} x}} + \frac{{I_{2} }}{{v_{0} - v_{p} }}e^{{ - iq_{2} x}} } \right)e^{i\omega t} .$$

In these expressions, $I_{\omega } \left( {x,t} \right) = - e\left( {v_{0} \delta n_{\omega } + n_{0} \delta v_{\omega } } \right)W$ is the plasmonic current in a channel of width $W$, while $V_{\omega } \left( {x,t} \right) = \delta \varphi_{\omega }$ is the voltage distribution within the plasma wave. The wavevectors $q_{1,2} = \omega /\left( {v_{0} \pm v_{p} } \right)$ describe acoustic plasma waves propagating with ($q_{1}$) and opposite to ($q_{2}$) the electron drift, at respective velocities $v_{0} + v_{p}$ and $v_{0} - v_{p}$. $v_{p} = \sqrt {e^{2} n_{01} /m^{*} C}$ is the velocity of the acoustic plasmons in the gated 2D channel in the absence of drift^29^ and the constant coefficients $I_{1}$ and $I_{2}$ are determined by the boundary conditions. The gated sections of the transistor channel serve as a waveguide for the plasma waves and can be represented by an equivalent-circuit diagram (Fig. 1d) comprising a transmission line (TL) with distributed capacitance ${\mathcal{C}}_{1} = CW$, kinetic inductance ${\mathcal{L}}_{1} = m^{*} /(e^{2} n_{01} W)$, and (if scattering is included) resistance ${\mathcal{R}}_{1} = {\mathcal{L}}_{1} /\tau$ (all defined per unit channel length)^30,31^. In the ungated sections of the transistor channel (where $n_{02} \gg n_{01}$) the plasma eigenfrequencies are well separated from those in the gated sections (if both sections are of comparable length). Consequently, we are able to restrict our analysis to frequencies close to those of the gated plasmons. In this limit, plasma oscillations in the ungated regions of the channel, having much higher frequency, are not excited. In our equivalent-circuit diagram (Fig. 1d), we therefore represent the ungated sections of the channel by a lumped inductance ${\mathcal{L}}_{2} = m^{*} L_{2} /e^{2} n_{02} W$ and resistance ${\mathcal{R}}_{2} = {\mathcal{L}}_{2} /\tau$, yielding total impedance $Z_{u} = {\mathcal{R}}_{2} + i\omega {\mathcal{L}}_{2}$. In the limit $\omega \tau \gg 1$, we use $Z_{u} \approx i\omega {\mathcal{L}}_{2}$ in our calculations.

The boundary between gated and ungated sections of the channel represents a region of special interest in this work. While a hydrodynamic description of plasma waves in a uniform 2D electron channel is completely equivalent to that involving the TL telegrapher’s equations, the boundary between two different regions requires additional modeling. As shown in^27^, at the boundary between gated and ungated sections of the transistor channel, some part of the ac plasmonic current induced by the plasma wave flows into the gate, via the fringing capacitance between its edge and the ungated section of the channel. We also assume continuity of the electric potential across the boundary between the gated and ungated regions, something that is justified if the length of the transition region is smaller than the electron–electron scattering length (*i.e*., if ballistic transport across this boundary is assumed^23^). Therefore, for plasma waves propagating throughout the channel, the boundary between gated and ungated regions can be modeled using the shunt capacitances $C_{b1,2}$ shown in Fig. 1d. In this way we account for the current that leaks from the channel into the gate, and also include the phase shift between incoming and reflected plasma waves^32,33^, caused by the reactive (capacitive) nature of the terminating impedances at the edges of the gated plasmonic cavity. While this model does not describe edge plasmonic modes, localized near the boundaries^34^, we note that these modes do not contribute to the plasmonic bands as their localization length should be much smaller than the lattice constant of the plasmonic crystal. The influence of the edge modes on the boundary conditions may be important at sharp (step-like) boundaries^34^, for which the transition region between gated and ungated sections of the channel will be much shorter than the localization length of any edge plasmons. This condition is not fulfilled in our system, in which the length of the transition region is limited at least by the thickness of the gate dielectric layer (typically tens of nanometers). On the other hand, the boundary conditions we impose are based on continuity of the electric potential and conservation of total current and are mostly insensitive to the details of the transition-region geometry, as long as ballistic electron transport is maintained across the boundary^23^.

The value of the fringing capacitance between the gate edge and the adjacent ungated section of the channel can be estimated as $C_{b1} = \alpha \varepsilon \varepsilon_{0} W$, where $\alpha = \left( {1/\pi } \right){\text{ln}}\left( {\pi L_{2} /d} \right)\sim 1$ is a geometric factor^27^. Due to the deliberate asymmetry that we create by placing an additional narrow metal finger on one side (the left) of the wide gate (see Fig. 1a and c), the fringing capacitance $C_{b2}$ at this edge increases: $C_{b2} = \gamma C_{b1}$ where $\gamma > 1$, see estimates for $\gamma$ in^35^.

The generic plasma dispersion equation for the 1D plasmonic crystal can be written using the Bloch theorem and the transfer matrix $\hat{T}$ connecting the values of $I_{\omega }$ and $V_{\omega }$ at opposite sides of the crystal elementary cell^19^:

3 $$det\hat{T} - e^{ikL} Tr\hat{T} + e^{2ikL} = 0,$$

where $k \in \left[ { - \pi /L,\pi /L} \right]$ is the Bloch wavevector and $L = L_{1} + L_{2}$ is the length of the elementary cell (see Fig. 1(a)). The transfer matrix for the gated section ($\hat{t}_{g}$) found from (2) is:

4 $$\hat{t}_{g} = e^{ - iM\theta } \left( {\begin{array}{*{20}c} {\cos \theta - iM\sin \theta } & {iZ_{0} \sin \theta } \\ {\frac{i}{{Z_{0} }}\left( {1 - M^{2} } \right)\sin \theta } & {\cos \theta + iM\sin \theta } \\ \end{array} } \right),$$

where $\theta = \omega L_{1} /\left( {v_{p} \left( {1 - M^{2} } \right)} \right)$, $Z_{0} = 1/CWv_{p}$ is the characteristic impedance of the plasmonic TL^28^ and $M = v_{0} /v_{p}$ is the Mach number in the electron fluid. Transfer matrices for the ungated section ($\hat{t}_{u}$) and the boundary between gated and ungated sections ($\hat{t}_{b}$) are:

5 $$\hat{t}_{u} = \left( {\begin{array}{*{20}c} 1 & {Z_{u} } \\ 0 & 1 \\ \end{array} } \right) , \hat{t}_{b} \left( {Z_{b1,2} } \right) = \left( {\begin{array}{*{20}c} 1 & 0 \\ {\frac{1}{{Z_{b1,2} }}} & 1 \\ \end{array} } \right),$$

where $Z_{b1,2} = 1/i\omega C_{b1,2}$. The transfer matrix $\hat{T}$ can be written in terms of the matrices in (4) and (5):

6 $$\hat{T} = \hat{t}_{g} \hat{t}_{b} \left( {Z_{b1} } \right)\hat{t}_{u} \hat{t}_{b} \left( {Z_{b2} } \right).$$

By substituting (6) into (3) we obtain the plasma dispersion equation:

7 $$\cos \left( {kL + M\theta } \right) = \left[ {1 + \frac{{Z_{u} }}{2}\left( {\frac{1}{{Z_{b1} }} + \frac{1}{{Z_{b2} }}} \right)} \right]\cos \theta + \frac{i}{2}\left[ {Z_{0} \left( {\frac{1}{{Z_{b1} }} + \frac{1}{{Z_{b2} }}} \right) + \frac{{Z_{0} Z_{u} }}{{Z_{b1} Z_{b2} }} + MZ_{u} \left( {\frac{1}{{Z_{b1} }} - \frac{1}{{Z_{b2} }}} \right) + \left( {1 - M^{2} } \right)\frac{{Z_{u} }}{{Z_{0} }}} \right]\sin \theta .$$

In the next section this equation is solved numerically, allowing the conditions for plasma-wave instability to be found.

### Simulation results

**Figure 2:**
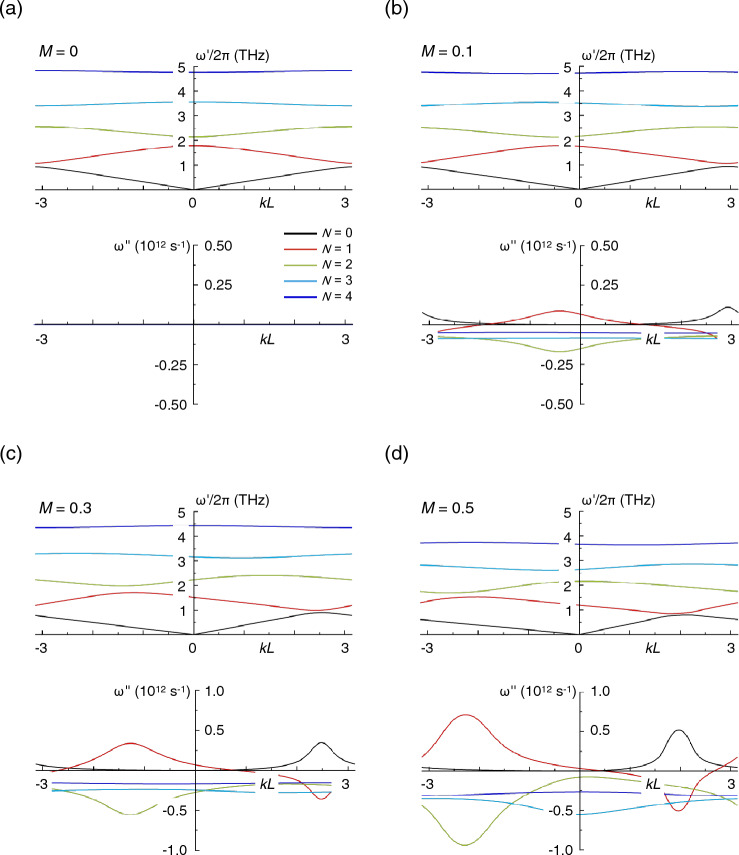
Variation of real (upper panel) and imaginary (lower panel) components of the plasma frequency for various values of the Mach number (*M*) and for *N* = 0, 1, 2, 3 & 4 (see lower panel of (**a**) for legend defining correspondence to lines colors). (**a**) *M* = 0. (**b**) *M* = 0.1. (**c**) *M* = 0.3. (**d**) *M* = 0.5. Here, $N$ is the plasmonic band index with $N = 0$ corresponding to the acoustic plasmonic crystal mode.

We have solved (7) numerically and found complex plasma frequency ($\omega = \omega^{\prime} + i\omega ^{\prime\prime}$) as a function of the Bloch vector $k$. The real part of $\omega$ corresponds to the frequency ($\omega^{\prime}/2\pi$) of the plasma wave. The imaginary part, on the other hand, determines the instability increment ($\omega ^{\prime\prime} < 0$) or decrement ($\omega ^{\prime\prime} > 0$), that is the growth or decay of the plasma wave, respectively, as it propagates while reflecting back and forth across the relevant region. In Fig. 2, we plot the calculated dispersions $\omega^{\prime}\left( k \right)/2\pi$ and $\omega ^{\prime\prime}\left( k \right)$, for the first five plasmonic bands and for four different values of the Mach number (panels (a)–(d)). Motivated by experiments such as that of^16^, we consider an InGaAs-based TeraFET ($m^{*} =$ 0.042 $m_{o}$, $m_{o} = 9.11 \times 10^{ - 31}$ kg is the free-electron mass; $\varepsilon = 13$) with $L_{1} = L_{2} = 200$ nm, $n_{01} =$ 3 × 10^11^ cm^-2^, $n_{02} /n_{01}$ = 3, and $d/L =$ 0.05. We also assume that the narrow metal finger positioned on the left side of each wide gate increases the fringing capacitance so that $C_{{b_{2} }} /C_{{b_{1} }} = \gamma >$ 1. The parameter $\gamma$ therefore characterizes the asymmetry of the boundary conditions at the opposite edges of each of the gate-defined plasmonic cavities. In the calculations here we assume $\gamma = 2$, a reasonable value that should be readily attainable in experiment.

In Fig. 2a, we show the real (upper panel) and imaginary (lower panel) components of $\omega$ when no dc drift is present in the system (*i.e*., $M =$ 0). The figure plots the first five plasmonic bands ($N =$ 0–4) generated by the coupling among the different cells, where the solution for $N = 0$ corresponds to the gapless acoustic mode of the plasmonic crystal for which $\omega \to 0$ at $k \to 0$. The lower panel shows that $\omega^{\prime\prime} = 0$ for all of the modes, a general result when $M =$ 0. In other words, no instability or additional damping of the plasma waves occur for this condition. The situation changes dramatically when a dc current is passed through the transistor ($M > 0$, panels (b)–(d)). All modes now develop a non-zero imaginary component of their plasma frequency, with $\omega ^{\prime\prime} < 0$ for all modes except for the acoustic one (and partially for the $N = 1$ mode). The negative sign of $\omega ^{\prime\prime}$ corresponds to instability and to exponential growth ($\sim e^{{\left| {\omega ^{\prime\prime}} \right|t}}$) of the plasma waves with increment $\left| {\omega ^{\prime\prime}} \right|$. The growth occurs due to the dissimilar reflection experienced by the drifting plasma waves at the asymmetric boundaries of each gated cavity. While this situation holds when the drift is from the boundary with low terminal impedance to that with high impedance (*i.e.*, when $M > 0$), reversing the direction of the drift results in attenuation of the plasma waves^8^. This result also follows from the form of (7), which implies that drift velocity reversal ($M < 0$) is equivalent to changing $\omega ^{\prime\prime}$ to $- \omega ^{\prime\prime}$, *i.e.,* that the plasma wave increment is converted to a decrement.

The results in Fig. 2b–d demonstrate that $\omega ^{\prime\prime}$ systematically becomes more negative with increasing dc bias corresponding to larger $M$. Although not included here, our numerical analysis shows that $\omega ^{\prime\prime}$ reaches its most negative values at $M\sim$ 0.9, before dropping back towards zero at $M \to$ 1. In the results presented in Fig. 2, we restrict our analysis to $M \le$ 0.5, reflecting the fact that the maximum drift achievable in practice is limited by velocity saturation.

It is evident from the results of Fig. 2 that $\omega ^{\prime\prime}$ varies in complicated fashion across the Brillouin zone, in a manner strongly dependent upon the band index $N$, a consequence of the changing symmetry of the coupled (quantized) modes in gate-defined cavities. In Fig. 2, $\omega^{\prime\prime} < 0$ in the entire Brillouin zone for all bands with $N \ge 2$. The instability increment $\left| {\omega ^{\prime\prime}} \right|$ is maximal for $N = 2$ and it is this mode that will therefore dominate the plasmonic response of the transistor. From inspection of the upper panel of Fig. 2d, we see that for this band the real part of $\omega$ corresponds to a plasma frequency of around 2 THz, pointing to the promise of this system for the generation of THz signals. While the instability should occur for any value of $k$ within the Brillouin zone, it is nonetheless strongest at some particular wavevector (whose value depends upon the Mach number). Since the Bloch vector determines the phase difference between oscillations in different elementary cells of the crystal, this result indicates the importance of phase matching of the unstable plasma oscillations in the different plasmonic cavities^23^.

**Figure 3:**
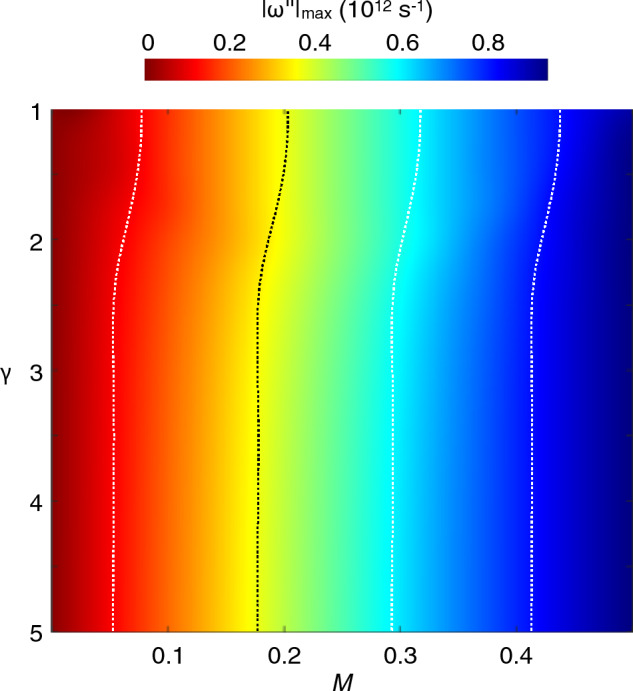
Two-dimensional contour showing the variation of the maximum plasma-wave increment $\left| {\omega ^{\prime\prime}} \right|_{max}$ as a function of $\gamma$ and *M*. Dotted contour lines serve as a guide to the eye to exhibit the dependence of $\left| {\omega ^{\prime\prime}} \right|_{max}$ on $\gamma$.

The plasma-wave increment also depends on the asymmetry factor ($\gamma$) of the array, as we explore in Fig. 3. Here we show the variation of the maximum value of the instability increment $\left| {\omega ^{\prime\prime}} \right|_{max}$ as a function of $\gamma$ and $M$. The contour indicates that $\left| {\omega ^{\prime\prime}} \right|_{max}$ increases as $\gamma$ is increased from 1 to around 3, following which it appears to saturate. This suggests that the influence of the asymmetric boundary conditions on the instability increment essentially reaches the limiting value, expected for ideally asymmetric boundaries (*i.e*., for $\gamma = \infty$^8^) once $\gamma =$ 3. This is a very encouraging result, as an asymmetry factor of $\gamma =$ 3 should be readily attainable by established nanolithography techniques.

We should also point out that the instability persists in the symmetric limit at $\gamma = 1$ once $M \ge$ 0.1. This result has been presented and discussed in^35^. However, in this case the instability only develops over some narrow ranges of the Bloch wave vector and therefore requires very precise phase-matching conditions between plasma oscillations in the elementary cells, something that is possible only for near-perfect gate periodicity. The plasma instability in symmetric arrays was first considered in^23^, and our results at $\gamma = 1$ are consistent with the findings of this paper. The structural asymmetry that we introduce here, on the other hand, triggers the DS instability in the individual elementary cells of the array, amplified by the coherent behavior of the unit cells in the plasmonic crystal. Since this occurs across the entire Brillouin zone (see Fig. 2) it should render demonstration of the instability easier in practice, where perfect gate periodicity is difficult to maintain. In the asymmetric limit, the instability increments are notably larger than those obtained in the symmetric one. Also, in this limit there is no the drift velocity threshold for the instability, which starts at any $M > 0$ as long as the increment exceeds the collisional damping.

## Discussion

In order for the plasmonic effects that we have discussed to be observed in practice, there are important experimental conditions that should be satisfied. The first set of these relate to the characteristics of the semiconductor heterostructure in which the plasmonic crystal is implemented. Specifically, in order to ensure sustained plasma oscillations, we require that $\omega^{\prime}\tau \gg 1$, where $1/\tau$ is the momentum relaxation rate due to phonon and impurity scattering. In addition, to realize coherent growth of the plasma oscillations, the condition $\left| {\omega ^{\prime\prime}} \right| > 1/2\tau$ should also be met^8^. Referring to the data for $M =$ 0.5 in Fig. 2d, we identify $\omega^{\prime}/2\pi$ $\sim$ 2.1 × 10^12^ Hz and $\omega^{\prime\prime}$ $\sim$ 10^12^ s^−1^. Based on these values, we therefore require $\tau$ > $\sim$ 0.5 ps, a value that should be readily achievable in InGaAs heterostructures at room temperature^16^. The properties of the semiconductor channel also impose limitations on the maximum dc bias that can be applied to the plasmonic crystal, as this bias should not exceed the value associated with spontaneous optical-phonon emission in the material. Even for the largest bias considered in Fig. 2d (corresponding to $M = 0.5$), the drift velocity $v_{0} \approx 3 \times 10^{5}$ m/s, less than the saturation threshold for this material^36^. At the same time, at an average mobility $\mu = 2 \times 10^{4}$ cm^2^ /Vs, the required drain-voltage drop across each plasmonic cavity will be around 30 mV, below the level at which hot-electron effects can be expected to impact transistor performance.

The second set of considerations relate to the lithography requirements associated with the implementation of the plasmonic gate arrays. In Fig. 1c, we demonstrate our capacity of using high-resolution electron-beam lithography to implement the proposed asymmetric grating gate array. This structure was fabricated on a semi-insulating GaAs substrate, without any two-dimensional electron channel, simply to demonstrate the feasibility of the array fabrication. The fidelity of the fabricated structure shown here is clearly high, and meets the requirements of our theory, with a high degree of reproducibility from one unit cell to the next. The Cr/Au (5–50 nm) metal lines were fabricated via a standard lift-off process, using a bilayer PMMA resist combination (PMMA 495 A4/PMMA 950 A2, total resist thickness of $\sim$ 250 nm), with careful optimization of the electron-beam proximity correction, exposure dose, and development time required in order to achieve the desired gate geometry. Nonetheless, for the purpose of a laboratory-scale demonstration of the TeraFET, these results establish the viability of realizing the physical dimensions required to implement this concept. For eventual wafer-scale manufacture of these devices, the advanced tools available to industry (such as extreme-UV lithography) should enable them to be readily fabricated.

In conclusion, we have proposed a design for a TeraFET with an asymmetric gate-array structure, which should be realizable in conventional III-V-based heterostructure systems. Central to the design of these devices is the introduction of deliberate asymmetry, which is implemented through the inclusion of a narrow gate that serves to break the symmetry within the basic cell of the plasmonic crystal. We have demonstrated that the DS instability in the current-biased transistor is dramatically amplified in the asymmetric elementary cells, reflecting the coherence of plasma oscillations across the entire crystal. By calculating the energy spectrum of the plasmonic crystal and its instability increments, we analyzed the dependence of the latter on the electron drift velocity and the extent of the structural asymmetry. Our results point to the possibility of exciting radiating steady-state plasma oscillations at the instability endpoint at room temperature, in arrays whose gate asymmetry should be readily implementable via standard nanolithography (as in Fig. 1c). We therefore consider this approach to represent a promising pathway to the generation of THz signals, for applications such as wireless networks-on-chip^1^ and beyond-6G systems.

### Supplementary Information

Supplementary Information.

## Data Availability

The data that support the findings of this study are available from the corresponding author upon reasonable request.

## References

[1] Akyildiz IF, Jornet JM, Han C (2014). Terahertz band: Next frontier for wireless communications. Phys. Commun..

[2] Yin XX, Baghai-Wadji A, Zhang Y (2022). A biomedical perspective in terahertz nano-communications—a review. IEEE Sens. J.urnal.

[3] Maestrini A (2012). Design and characterization of a room temperature all-solid-state electronic source tunable from 2.48 to 2.75 THz. IEEE Trans. Terahertz Sci. Technol..

[4] Acharyya A, Banerjee JP (2014). Prospects of IMPATT devices based on wide bandgap semi-conductors as potential terahertz sources. Appl. Nanosci..

[5] Asada M, Suzuki S (2021). Terahertz emitter using resonant-tunneling diode and applications. Sensors.

[6] Sengupta K, Nagatsuma T, Mittleman DM (2018). Terahertz integrated electronic and hybrid electronic–photonic systems. Nat. Electron..

[7] Shur M, Aizin G, Otsuji T, Ryzhii V (2021). Plasmonic field-effect transistors (TeraFETs) for 6G communications. Sensors.

[8] Dyakonov M, Shur M (1993). Shallow water analogy for a ballistic field effect transistor: new mechanism of plasma wave generation by dc current. Phys. Rev. Lett..

[9] Bhardwaj S, Nahar NK, Rajan S, Volakis JL (2016). Numerical analysis of Terahertz emissions from an ungated HEMT using full-wave hydrodynamic model. IEEE Trans. Electron Devices..

[10] Li K, Hao Y, Jin X, Lu W (2017). Hydrodynamic electronic fluid instability in GaAs MESFETs at terahertz frequencies. J. Phys. D: Appl. Phys.

[11] Nafari M, Aizin GR, Jornet JM (2018). Plasmonic HEMT terahertz transmitter based on the Dyakonov–Shur instability: performance analysis and impact of nonideal boundaries. Phys. Rev. Appl..

[12] Mendl CB, Polini M, Lucas A (2021). Coherent Terahertz radiation from a nonlinear oscillator of viscous electrons. Appl. Phys. Lett..

[13] Crabb J, Cantos-Roman X, Jornet JM, Aizin GR (2021). Hydrodynamic theory of the Dyakonov–Shur instability in graphene transistors. Phys. Rev. B.

[14] Knap W (2004). Terahertz emission by plasma waves in 60-nm gate high electron mobility transistors. Appl. Phys. Lett..

[15] El Fatimy A (2010). AlGaN/GaN high electron mobility transistors as voltage-tunable room temperature terahertz sources. J. Appl. Phys..

[16] Barut B (2022). Asymmetrically engineered nanoscale transistors for on-demand sourcing of terahertz plasmons. Nano Lett..

[17] Popov VV (2014). Detection of terahertz radiation by tightly concatenated InGaAs field-effect transistors integrated on a single chip. Appl. Phys. Lett..

[18] Allen SJ, Tsui DC, Logan RA (1977). Observation of the two-dimensional plasmon in silicon inversion layers. Phys. Rev. Lett..

[19] Aizin GR, Mikalopas J, Shur M (2020). Plasmonic instabilities in two-dimensional electron channels of variable width. Phys. Rev B.

[20] Krasheninnikov MV, Chaplik AV (1981). Two-dimensional plasma waves in superlattices. Sov. Phys. Semicond..

[21] Mackens U, Heitman D, Prager L, Kotthaus JP, Beinvogl W (1984). Minigaps in the plasmon dispersion of a two-dimensional electron gas with spatially modulated charge density. Phys. Rev. Lett..

[22] Wilkinson RJ (1992). Plasmon excitation and self-coupling in a biperiodically modulated two-dimensional electron gas. J. Appl. Phys..

[23] Petrov AS, Svintsov D, Ryzhii V, Shur M (2017). Amplified-reflection plasmon instabilities in grating-gate plasmonic crystals. Phys. Rev. B..

[24] Otsuji T (2008). Emission of terahertz radiation from dual grating gate plasmon-resonant emitters fabricated with InGaP/InGaAs/GaAs material systems. J. Phys. Condens. Matter.

[25] Mönch, E. S. *et al.* Nonlinear intensity dependence of ratchet currents induced by terahertz laser radiation in bilayer graphene with asymmetric periodic grating gates. Preprint at https://arxiv.org/abs/2306.15405.

[26] Boubanga-Tombet S (2020). Room-temperature amplification of terahertz radiation by grating-gate graphene structures. Phys. Rev. X.

[27] Aizin GR, Mikalopas J, Shur M (2023). Plasma instability and amplified mode switching effect in THz field effect transistors with a grating gate. Phys. Rev B.

[28] Aizin GR, Mikalopas J, Shur M (2016). Current-driven plasmonic boom instability in three-dimensional gated periodic ballistic nanostructures. Phys. Rev. B.

[29] Chaplik AV (1972). Possible crystallization of charge carriers in low-density inversion layers. Sov. Phys. JETP.

[30] Burke PJ, Spielman IB, Eisenstein JP, Pfeiffer LN, West KW (2000). High frequency conductivity of the high-mobility two-dimensional electron gas. Appl. Phys. Lett..

[31] Aizin GR, Dyer GC (2012). Transmission line theory of collective plasma excitations in periodic two-dimensional electron systems: Finite plasmonic crystals and Tamm states. Phys. Rev. B.

[32] Rejaei B, Khavasi A (2015). Scattering of surface plasmons on graphene by a discontinuity in surface conductivity. J. Opt..

[33] Jiang BY, Mele EJ, Fogler MM (2018). Theory of plasmon reflection by a 1D junction. Opt. Express.

[34] Siaber S, Zonetti S, Cunningham JE, Sydoruk O (2019). Terahertz plasmon resonances in two-dimensional electron systems: modeling approaches. Phys. Rev. Appl..

[35] Aizin, G. R., Mundaganur, S., Mundaganur, S. & Bird, J. P. Supplementary Information at 10.1038/s41598-024-62492-3.

[36] Quay R, Moglestue C, Palankovski V, Selberherr S (2000). A temperature dependent model for the saturation velocity in semiconductor materials. Mater. Sci. Semicond. Process..

