# Supplementary material for: Terahertz-frequency plasmonic-crystal instability in field-effect transistors with asymmetric gate arrays
Source: Sci Rep. 2024 May 24;14:11856. doi: 10.1038/s41598-024-62492-3 (PMC11126616; doi:10.1038/s41598-024-62492-3)
Supplement: Supplementary file 1 — Supplementary Information. [file 41598_2024_62492_MOESM1_ESM.docx]

**Supplemental Information**

1. **Estimates of the fringing capacitance**

The fringing capacitance $C_{b1}$ between the gate and adjacent ungated 2D electron channel was estimated in Ref. [27] of the main text as

$C_{b1}=\alpha\varepsilon\varepsilon_{0}W , \alpha=\frac{1}{\pi}\ln\frac{\pi L_{2}}{d}$ , (S1)

where $W$ is the transistor width, $L_{2}$ is the length of the ungated channel (cutoff length), and $d$ is the separation distance between the channel and the gate as shown in Fig. S1(a). Placing a narrow metal finger at some distance $d_{1}$ from the wide gate increases the total fringing capacitance, $C_{b2}$, by adding additional capacitive links between the gate and the channel through the metal finger. These links are described by capacitances $C_{1}$ and $C_{2}$ indicated in Fig. S1(a) (see also Fig. 1(d) of the main paper).


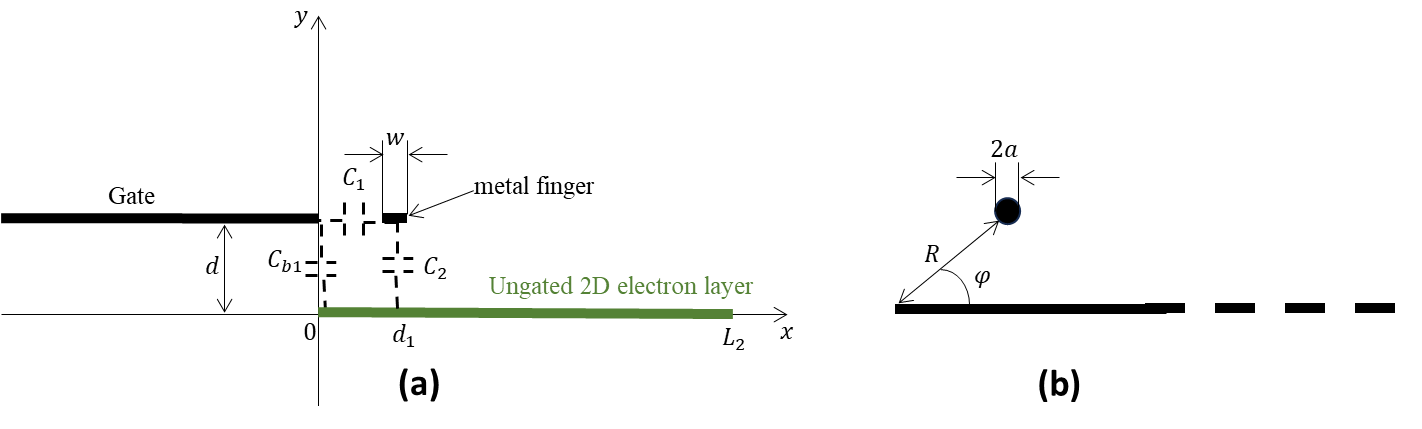


**Fig. S1.** (a) The structure geometry near the boundary between gated and ungated parts of the transistor channel in the presence of an additional metal finger. Capacitive links between different elements of the structure are shown by dashed lines. (b) The wire-over-half-plane geometry used in the evaluation of the capacitance in Eq. (S2).

In calculations of the capacitances $C_{1}$ and $C_{2}$ one should take into account that the thickness of the metal layer forming the finger is comparable with its width $w$ ($w\sim$ 25-50 nm), allowing the finger to be modelled as a metal wire of radius $a=w/2$. Then, capacitances $C_{1}$ and $C_{2}$ can be evaluated using a standard result for the capacitance between a wire and a half-plane, as shown in Fig. S1(b) [1]

$C=\frac{2\pi\varepsilon\varepsilon_{0}W}{\ln\left( \frac{4R\sin\varphi/2}{a} \right)}$ . (S2)

The last formula is applicable for $R\gg a$ but given a weak logarithmic dependence of the results on spatial dimensions of this system it can be used for numerical estimates. Capacitances $C_{1}$ and $C_{2}$ are described by Eq. (S2) with $R=d_{1}$, $\varphi=\pi$ and $R=\sqrt{d_{1}^{2}+d^{2}}$ , $\varphi=\tan^{-1} \left( d/d_{1} \right)$, respectively.

The capacitance $C_{b2}$ can be evaluated as

$C_{b2}=C_{b1}+\frac{C_{1}C_{2}}{C_{1}+C_{2}}$ . (S3)

Using expressions for $C_{b1}$, $C_{1}$, and $C_{2}$ from Eqs. (S1) and (S2) in Eq. (S3) we obtain

$C_{b2}=\gamma C_{b1} , \gamma=1+\frac{2\pi}{\alpha}\left[ \ln\left( \frac{32\sqrt{2}d_{1}\left[ d^{2}+d_{1}^{2}-d_{1}\sqrt{d_{1}^{2}+d^{2}} \right]^{1/2}}{w^{2}} \right) \right]^{-1}$. (S4)

The last equation defines the geometric factor $\gamma$ used in the main text. Numerical estimates made for the transistor structure shown in Fig. 1(c) yield $\gamma\sim2-6$.

**2. Plasmonic band spectrum in current-driven transistor structures with symmetric grating gate arrays**

The developed theory can be used for calculation of the plasmonic band spectrum in the current-biased transistor structures with symmetric grating gate, i.e. in the absence of an additional metal finger. This limit is described by Eq. (7) with symmetry factor $\gamma=1$. The results are shown in Fig. S2.

In this calculation, we kept the values of all material and geometric parameters the same as in Fig. 2 of the main text. These results qualitatively reproduce the results obtained in [23] where the plasmonic band spectrum in the current-biased 2D electron channels with symmetric grating gate was first calculated. It follows from these results that plasma instability is still possible in the symmetric limit, but that it only occurs over some narrow ranges of the Bloch wave vector when proper phase-matching conditions between plasma oscillations in individual elementary cells are satisfied [23]. Experimental observation of this type of instability requires near-perfect gate periodicity which is difficult to maintain in multi-gated structures with many ($N\sim$10^2^) unit cells. On the other hand, in the multi-gated structures with structural asymmetry suggested in this paper, the DS plasma instability develops in the respective unit cells, which are coupled coherently in the plasmonic crystal. As a result, instability occurs in the entire Brillouin zone and its experimental observation is less sensitive to small deviations from perfect gate periodicity.


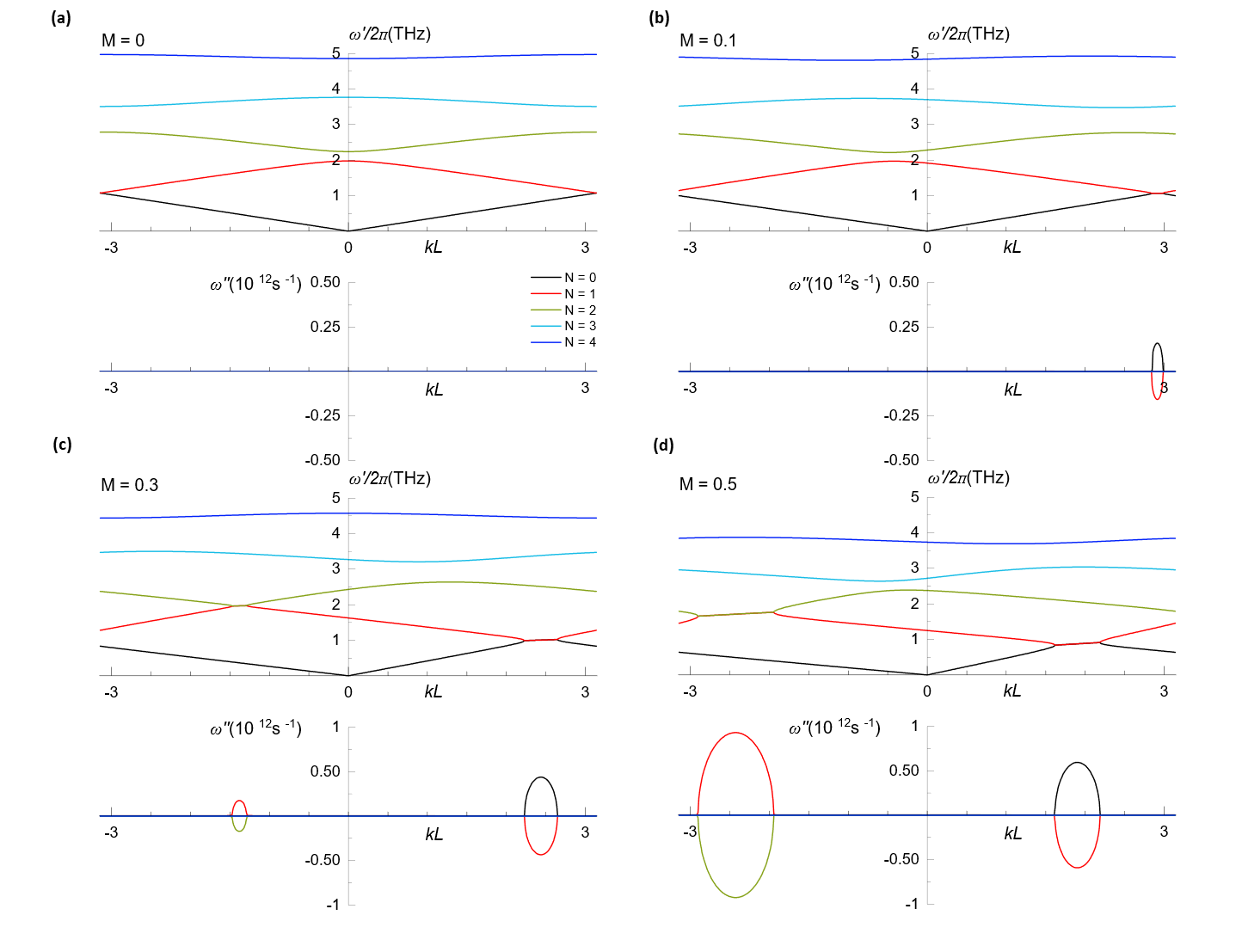


**Fig. S2.** Variation of real (upper panel) and imaginary (lower panel) components of the plasma frequency in transistor structures with symmetric grating gate arrays for various values of the Mach number *M*. (a) *M* = 0. (b) *M* = 0.1. (c) *M* = 0.3. (d) *M* = 0.5. Here, $N$ is the plasmonic band index.

[1] Iossel’, Yu. A., Kochanov, E. S. & Strunskiy, M. G. The Calculation of Electrical Capacitance, p.92 (U.S. Air Force Foreign Technology Division, 1971). http://apps.dtic.mil/sti/tr/pdf/AD0727198.pdf
